# Markers of Oxidative Stress in the Exhaled Breath Condensate of Workers Handling Nanocomposites

**DOI:** 10.3390/nano8080611

**Published:** 2018-08-10

**Authors:** Daniela Pelclova, Vladimir Zdimal, Jaroslav Schwarz, Stepanka Dvorackova, Martin Komarc, Jakub Ondracek, Martin Kostejn, Petr Kacer, Stepanka Vlckova, Zdenka Fenclova, Alexey Popov, Lucie Lischkova, Sergey Zakharov, Dhimiter Bello

**Affiliations:** 1 Department of Occupational Medicine, First Faculty of Medicine, Charles University in Prague and General University Hospital in Prague, Na Bojisti 1, 128 00 Prague 2, Czech Republic; Stepanka.Vlckova@vfn.cz (S.V.); zdenka.fenclova@lf1.cuni.cz (Z.F.); Lucie.Lischkova@vfn.cz (L.L.); Sergej.Zacharov@vfn.cz (S.Z.); 2 Institute of Chemical Process Fundamentals of the CAS, Rozvojová 1/135, 165 02 Prague 6, Czech Republic; zdimal@icpf.cas.cz (V.Z.); schwarz@icpf.cas.cz (J.S.); ondracek@icpf.cas.cz (J.O.); kostejn@icpf.cas.cz (M.K.); 3 Department of Machining and Assembly, Department of Engineering Technology, Department of Material Science, Faculty of Mechanical Engineering, Technical University in Liberec, Faculty of Mechanical Engineering, Studentská 1402/2, 461 17 Liberec, Czech Republic; stepanka.dvorackova@tul.cz (S.D.); alespopov@yandex.ru (A.P.); 4 Institute of Biophysics and Informatics, First Faculty of Medicine, Charles University in Prague and General University Hospital in Prague, Salmovská 1, 120 00 Prague 2, Czech Republic; martin.komarc@lf1.cuni.cz; 5 Faculty of Physical Education and Sport, First Faculty of Medicine, Charles University in Prague and General University Hospital in Prague, José Martího 31, 162 52 Prague 6, Czech Republic; 6 Biocev, 1st Faculty of Medicine, Charles University, Prumyslova 595, 252 50 Vestec, Czech Republic; petr.kacer69@gmail.com; 7 Department of Biomedical and Nutritional Sciences, Zuckerberg College of Health Sciences, Lowell, MA 01854, USA; Dhimiter_Bello@uml.edu

**Keywords:** nanoparticles, workers, nanocomposites, inhalation, exhaled breath condensate, oxidative stress, occupational exposure

## Abstract

Researchers in nanocomposite processing may inhale a variety of chemical agents, including nanoparticles. This study investigated airway oxidative stress status in the exhaled breath condensate (EBC). Nineteen employees (42.4 ± 11.4 y/o), working in nanocomposites research for 18.0 ± 10.3 years were examined pre-shift and post-shift on a random workday, together with nineteen controls (45.5 ± 11.7 y/o). Panels of oxidative stress biomarkers derived from lipids, nucleic acids, and proteins were analyzed in the EBC. Aerosol exposures were monitored during three major nanoparticle generation operations: smelting and welding (workshop 1) and nanocomposite machining (workshop 2) using a suite of real-time and integrated instruments. Mass concentrations during these operations were 0.120, 1.840, and 0.804 mg/m^3^, respectively. Median particle number concentrations were 4.8 × 10^4^, 1.3 × 10^5^, and 5.4 × 10^5^ particles/cm^3^, respectively. Nanoparticles accounted for 95, 40, and 61%, respectively, with prevailing Fe and Mn. All markers of nucleic acid and protein oxidation, malondialdehyde, and aldehydes C_6_–C_13_ were elevated, already in the pre-shift samples relative to controls in both workshops. Significant post-shift elevations were documented in lipid oxidation markers. Significant associations were found between working in nanocomposite synthesis and EBC biomarkers. More research is needed to understand the contribution of nanoparticles from nanocomposite processing in inducing oxidative stress, relative to other co-exposures generated during welding, smelting, and secondary oxidation processes, in these workshops.

## 1. Introduction

The global market for nanocomposites is expected to reach over $5 billion by 2020, and it is fueled by demand in numerous sectors, from automotive to military and aerospace applications [1]. Research and development in nanocomposite synthesis and processing proceed larger scale commercialization. Literally thousands of laboratories in academia and private industrial sector engage in nanocomposite research and development by experimenting with a variety of nanofillers and polymer types. Interest in understanding exposures to engineered nanomaterials used as fillers in nanocomposite synthesis, as well as incidental nanoparticle and carbon nanotube (CNT) exposure during nanocomposite synthesis and post-processing can be traced as far back as a decade ago [1,2]. Several recent studies have focused on end-of-life grinding and recycling of CNT-composites [3,4]. In contrast to numerous studies on exposures, little information is available on the health effects of workers and researchers engaged in nanocomposite research and development or manufacturing [5,6,7,8]. Polycyclic aromatic hydrocarbons (PAHs) are chemicals reactive towards a number of atmospheric oxidants. It should be noted that these environments are often characterized by mixed exposures (vapor and aerosols phases and mixed chemistry). Although the focus is on nanoparticles, other co-exposures may play an important role in the health effect. 

Volatile organic compounds (VOCs) and semi-volatile organic compounds (SVOCs), generated as a result of polymer heating and thermal degradation, polycyclic aromatic hydrocarbons (PAHs), and other byproducts of chemical reactions with ozone and catalytic metal oxides used as fillers, may lead to complex airborne pollutants. Lower molecular weight PAH vapor may be converted to higher molecular weight PAHs and other compounds via catalytic reactions with metal oxide fillers [9]. Condensation of organic matter on metal oxides nanoparticles may also enhance their transport in deep airways. Exposures to welding fumes that may take place alongside nanocomposite manufacturing include multiple transition metal species, especially redox active Zn and Mn species, both soluble and insoluble, and that exposures to Mn species vary with specific processes and shield gases [10]. Background ozone, as well as that produced by certain processes, such as welding, play an important role on secondary aerosol formation via oxidation reactions. Ozone is also a strong oxidant on its own right. With regards to ozone, the maximum allowable daily 8-h concentrations are not commonly exceeded [11]. The estimated percentage of the nanofraction of Mn deposited in a mild steel-welder’s respiratory system ranged between 10 and 56% [12]. For stainless steel welding, the nanoparticle respiratory deposition samplers collected 59% of the total Mn, 90% of the total Cr, and 64% of the total Ni. These results indicate that most of the Cr and more than half of the Ni and Mn in the fumes were in the fraction smaller than 300 nm [12]. Although our focus is on the lesser-studied exposures to nanoparticles, it is important to recognize that exposures at the nanocomposite synthesis and processing sites may be chemically more complex and less understood.

Since inhalation remains the major exposure pathway to nanoparticles during nanocomposite manufacturing, the respiratory system is the main portal of entry and the primary target organ of concern. However, there are only a few sensitive non-invasive methods to probe deep airways in humans. Exhaled breath condensate (EBC) collection and analysis is one such technique. EBC is a liquid that reflects the composition of the fluid lining the airway [13]. It is obtained non-invasively from subjects after cooling of the exhaled air. EBC is composed mainly of water (99.9%) and a small proportion of water-soluble and insoluble compounds. These non-volatile compounds can include small inorganic ions, large organic molecules (urea, organic acids, amino acids), proteins, and macromolecules that presumably originate from the airway-lining fluid in the form of aerosolized particles formed during the reopening of distal airways [14]. Analysis of EBC enables measurement of biomarkers present in the deep airways. 

A large body of in vivo and in vitro particle toxicology and nanotoxicology studies have shown that nanoparticles induce toxicity via a number of mechanisms, including intracellular reactive oxygen species (ROS) generation and ensuing oxidative stress, Ca^2+^ flux, induction of pro-inflammatory mediators through receptor stimulation, hypersensitivity, genotoxicity, and cell necrosis [15,16,17,18]. The homeostatic redox state of the host becomes disrupted upon ROS induction by nanoparticles. These sequential molecular and cellular events are known to cause oxidative stress, followed by severe cellular genotoxicity and then programmed cell death. Both experimental and epidemiological studies have indicated that chronic inflammation is involved in and plays a critical role in several chronic diseases, including respiratory and cardiovascular diseases, and lung tumorigenesis [17,19,20,21]. However, there is no evidence that particles below 100 nm show any step-change in their hazard meaning that there is no evidence of any novel “nano-specific hazard”. Therefore, conventional particle toxicology data are useful and relevant to the determination of the nanoparticle hazard [22]. 

A common feature of many aerosols in the workplaces is their ability to generate ROS, which induce oxidative damage to biomolecules, leading to activation of redox signaling pathways. 8-*iso*-Prostaglandin F2α (8-isoprostane) is produced by free-radical lipid peroxidation of arachidonic acid, and represents an in vivo specific marker of oxidative stress. Oxidative modification of lipids occurs in vivo during aging and in certain disease conditions. Lipid peroxides are unstable indicators of oxidative stress in cells that form more complex and reactive compounds, such as malondialdehyde (MDA), 4-hydroxy-*trans*-hexenal (HHE), and 4-hydroxy-*trans*-nonenal (HNE), that can form covalent adducts with biomolecules including DNA and proteins, and thus, are regarded as genotoxic and cytotoxic [23]. 3-Nitrotyrosine (3-NOTyr) and 3-chlorotyrosine (3-ClTyr) are stable products of peroxynitrite (ONOO^−^) and hypochlorous acid (HClO), respectively, with tyrosine residues of proteins, which may lead to functional relationship with the neutrophilic inflammation. Both 3-NOTyr and *o*-tyrosine (*o*-Tyr) have been found in patients with interstitial lung diseases, but studies on other biomarkers are limited. Oxidative damage to nucleic acids may be measured using 8-hydroxy-2-deoxyguanosine (8-OHdG) and 8-hydroxyguanosine (8-OHG) formed by oxidation of guanine from DNA and 5-hydroxymethyl uracil (5-OHMeU) from RNA [16,23].

The first aim of this study was to characterize workplace aerosols generated during synthesis and post-processing of nanocomposites, and associated operations, including machining of nanocomposite materials, smelting, and welding. Another aim was to measure markers of oxidative stress in the EBC of workers exposed to nanoparticles and to study their possible association with workplace environments. This is the first study to report on the respiratory health status on this cohort of researchers and applicators, using a suite of well-established biomolecular markers measured in the EBC. In a separate paper, we report on inflammatory leukotrienes (B4 and cysteinyl leukotrienes), pro-inflammatory cytokines and chemokines, such as tumor necrosis factor (TNF), interleukins (IL 5, IL 9), and their anti-inflammatory counterparts (lipoxins; IL 4, and IL 10), in the EBC of workers, as well as fractional exhaled NO, and lung function using spirometry [24]. 

## 2. Materials and Methods 

### 2.1. Facility and Operations Description

The research and development unit at a national research university is trying to develop a new thermoplastic or reactoplastic (thermoset) composite material that exhibits comparable performance characteristics with steel, with regard to its low thermal expansion, hardness, and resistance to surface scratching. This new nanocomposite material is intended for use in automated measuring devices of plastic materials, where metal instruments cannot be used because of measurement errors related to their higher expansion and the risk of scratching the surface of plastics. 

Researchers would normally perform three different operations: welding on metal surfaces, smelting of mixtures containing nanoadditives, and machining of the finished nanocomposite. The researchers were divided into two separate groups in two workshops. Welding and smelting took place together in workshop 1, located in the basement of the building, whereas composite machining took place in a second workshop located on the ground floor of the same building. Researchers would usually carry on their daily operations in the workshops for about 2 h, with the majority of the working day spent in their offices. The three operations would occur simultaneously in both workshops, and be performed by different individuals.

On the day of examination, the duration of each of these (simultaneous) operations lasted about 150 min, and the original source of a nanocomponent was amorphous colloidal nanoSiO_2_ in epoxide resins.

Welding on metal surfaces (workshop 1) was conducted on mild steel S355J2 using metal active gas (MAG) technology with Ar/CO_2_ mixed-copper coated wire (G3Si) and coated electrodes EB 121 (content in wt %: C 0.05, Si 0.4, Mn 0.8). The S355J2 steel content was (in wt %): Fe, 97.39; C, 0.24; Mn, 1.70; Si, 0.6; P, 0.035; S, 0.035). Also in workshop 1, an AlSi_9_Cu_3_ alloy (content in wt %: Al, 83.50; Si, 10.0; Fe, 0.8; Cu, 3.0; Mn, 0.55; Mg, 0.25; Cr, 0.15; Ni, 0.55; Zn, 1.2), mixed with modifying salts (NaCl, KCl, NaF), was smelted in the smelting oven at 760 °C, in a mold made of sand mixed with a bentonite filler.

Machining of surfaces of previously finished nanocomposite blocks, including milling, grinding, and polishing, was performed in workshop 2. Mixing of the fillers with matrices to prepare nanocomposites samples was performed about twice per month, and lasted about 2 h.

On the day of examination, five nanocomposite specimens were processed on workshop 2. Two samples contained epoxide resins with SiO_2_ fillers. One nanocomposite specimen contained 1.0% *w*/*w* amorphous colloidal nanoSiO_2_, whereas the second specimen contained crushed rice husks with 50% cellulose, 30% lignin, and up to 20.0% SiO_2_ as fillers. Three additional samples were geopolymers, first containing metakaolin mixed with NaOH only, and two others were filled with ash or basalt, each at 40% *w*/*w*. The formulations of nanocomposites differed over time, according to research aims. On other days, the operations may include thermoplast melting (polypropylene) and mixing with milled coconut fibers, composite materials production, or adding textile fibers, glass, etc.

### 2.2. Subjects

EBC samples were collected from the 19 nanocomposite-synthesizing and processing researchers (14 men, 5 women, all non-smokers) in September 2016. On the day of examination, eleven researchers were working in workshop 1 (welding and smelting), and seven in workshop 2 (machining of nanocomposites). The control group was composed of 19 subjects (13 men, 6 women, all non-smokers) from the same town, not employed in this plant nor occupationally exposed to dust or other health risks. 

Exposed and control subjects were administered a standardized questionnaire that collected information on personal and occupational history, medical treatments, dietary habits, smoking habits, and alcohol intake. Participants underwent a physical examination in another part of the building, followed by the collection of their EBC. Exclusion criteria for all subjects were history of tuberculosis, myocarditis, congenital heart disease, lung cancer, and recent fever and/or common cold symptoms. Nanocomposite researchers provided EBC both pre-shift (i.e., before 2.5 h exposure to aerosol in the workshops) and post-shift (i.e., after aerosol exposure in the workshop). For simplicity, we refer to these examinations as pre-shift and post-shift, even though the remainder of their total 8-h shift was spent in their offices. Among the workers, seven were working in the same workshop as usual, and the remaining twelve were working in both workshops during the previous weeks. The controls provided EBC only once at approximately the same time of day as researchers in the workshop floor. The pre-shift samples were used to study the subacute/chronic effect on the subjects resulting from exposures in previous days. Comparison of the pre-shift and post-shift samples was intended to evaluate the acute effect of exposure during the shift.

The study was conducted according to the Declaration of Helsinki. The Ethical Committee of the 1st Medical Faculty, Charles University, approved the study. All participants were informed of the study aim at least five days earlier, and signed an informed consent form before the study began.

### 2.3. Workplace Aerosol Measurements

Chemical and physical characterization of nanoparticles and aerosols was carried out using a set of real-time and size selective aerosol instruments. A Berner low-pressure impactor (BLPI, HAUKE GmbH, Gmunden, Austria) [25,26] was used to sample aerosol particles onto 10 stages corresponding to their aerodynamic diameter, covering the 25 nm–13.6 µm size range. The impactor samples were analyzed gravimetrically on an M5P balance (Sartorius GmbH, Goettingen, Germany, 1 µg resolution), followed by ion chromatography (IC) in a Dionex 5000 (Dionex Co., Sunnyvale, CA, USA), whose two-channel system enabled parallel determination of water soluble ions (both anions and cations) as described by Talbot et al. [27]. The impactors were placed about 3 m from the individual processes at a height of approximately 1.5 m.

The online real-time instrumentation included two standard aerosol spectrometers—a scanning mobility particle sizer SMPS 3936L (TSI Inc., Shoreview, MN, USA) and an aerodynamic particle sizer APS 3321 (TSI Inc., Shoreview, MN, USA), covering the size range of aerosol particles from 6 nm up to 20 µm. In addition, an ultrafine condensation particle counter UCPC 3025 (TSI Inc., Shoreview, MN, USA) was used to measure the total particle number concentration (10 nm–~1 µm) and three optical particle sizers OPS 3330 (TSI Inc., Shoreview, MN, USA) measuring number size distribution in the range 300 nm–10 µm. All instruments sampled for the whole duration of the current nanoparticle generation operations (welding, soldering, synthesis, nanocomposite machining). Background nanoparticle concentration was monitored before the operations started. The online instruments were placed about 1.5 m from the workers at a height of approximately 1.5 m. The details concerning the samples collected are shown in Supplementary Table S1.

A scanning electron microscope (SEM) Indusem, (TESCAN ORSAY HOLDING a.s., Brno, Czech Republic), equipped with energy-dispersive X-ray (EDX) spectroscopy (XFlash detector 5010, Bruker, Karlsruhe, Germany), was used for elemental analyses and EDX spectra acquisition at an accelerating voltage of 15 kV for 120 s. Carbon sputtering was used for deposition of a conducting layer (ca. 15 nm thick) onto the samples, to prevent charging. To cover a representative area and minimize the effects of possible heterogeneities, seven square areas containing aerosol with an edge length of 200 μm were scanned on each sample. Carbon, oxygen, and copper were excluded from the results as they were present in the substrates, superficial conducting layer, and supporting material. 

The data from EDX were recalculated to mass concentration using sulfur as internal reference, and sulfate mass concentration from IC recalculated to sulfur under the assumption that all sulfur in the sample was present as sulfates. Control recalculations were done using chlorine as reference and chloride mass concentration from IC, with very similar results as for sulfur.

### 2.4. Collection and Analysis of Oxidative Stress Markers in EBC

EBC samples were collected using an Ecoscreen Turbo DECCS device (Jaeger, Hochberg, Germany), that was equipped with a filter. All subjects breathed tidally through a mouthpiece connected to the condenser (−20 °C) while wearing a nose-clip. A minimum volume of exhaled air of 120 L was maintained (monitored via the EcoVent device by Jaeger, Wurzburg, Germany), and the time of collection was about 15 min [9]. All samples were immediately frozen and stored at −80 °C. 

A panel of oxidative stress markers derived from free radical oxidation of polyunsaturated fatty acids, nucleic acid bases, and proteins was analyzed after solid-phase extraction (SPE) by liquid chromatography-electrospray ionization-tandem mass spectrometry (LC-ESI-MS/MS) using deuterium-labelled internal standards, as previously described [28,29,30]. These biomarkers included lipid oxidation—MDA, HHE, HNE, aldehydes C_6_–C_13_, and 8-isoprostane; nucleic acids—8-OHG, 8-OHdG, and 5-OHMeU; and protein-*o*-Tyr, 3-ClTyr, and 3-NOTyr. The α-amylase concentration was monitored [13] to account for any sample contamination by saliva [31]. Electrical conductivity of EBC was measured as a reference indicator in EBC dilution to account for changes in respiratory solute concentration [32]. All samples were blinded to personnel involved.

### 2.5. Environmental Contamination

Air concentrations of SO_2_, O_3_, nitrogen oxides (NO*_x_*), particulate matter (PM)_2.5_ and PM_10_, recorded on an hourly basis, were taken from the monitoring data on the same days when the workers were examined. Air concentrations were obtained from the National Hydrometeorological monitoring system at the closest stationary monitoring station. The distance to the site of EBC collection was about 3.0 km. Environmental monitoring employed the following analytical methods: UV-fluorescence (SO_2_), chemiluminescence (NO*_x_*), UV-absorption (O_3_), and an optoelectronic method (PM). 

### 2.6. Statistical Analysis

Basic descriptive statistics (mean, median, confidence interval, standard deviation, skewness, and kurtosis) were computed for all variables, which were subsequently tested for normality using the Kolmogorov–Smirnov test. The chi-square test was used to compare frequency counts of demographic categorical variables (alcohol consumption) in groups of workers vs. controls. Differences in interval variables were tested using independent-group *t*-test (for normally distributed variables) and the Mann-Whitney *U* test (for non-normally distributed variables, such as markers of oxidative stress in EBC). The paired sample *t* test (or the Wilcoxon signed-rank test as a nonparametric alternative) was used to compare workers’ pre-shift and post-shift values of oxidative stress markers. The bivariate relationship between variables under study was assessed using the Spearman correlation coefficient. Multiple regression analysis was used to predict markers in EBC by a set of predictors (nanoparticle exposure-yes/no, age, sex, alcohol consumption-yes/no, body mass index-BMI). Statistical significance was set at *p* < 0.05. All analyses were conducted using SPSS version 22.0 (SPSS, Inc., Chicago, IL, USA).

## 3. Results

### 3.1. Subjects

Demographic characteristics of the exposed and control groups are presented in Table 1. There were no differences in age, gender, body mass index, and alcohol consumption in the two groups studied (all *p* > 0.05).

Personal protective equipment was used for welding (welding helmets, leather gloves and leather aprons) and smelting (gloves). No respiratory protection was used during any of the operations.

### 3.2. Workplace Aerosol Results

#### 3.2.1. Number Size Distributions 

##### Metal Active Gas (MAG) Welding 

MAG welding resulted in monomodal particle size distribution, with particles mainly in the accumulation size range, with modal size around 200 nm. Total median particle number concentration was 1.3 × 10^5^ particles/cm^3^ (the interquartile range (IQR) was from 1.2 × 10^5^ to 1.5 × 10^5^ particles/cm^3^) and maximum number concentration reached about 2.1 × 10^5^ particles/cm^3^. However, these particles were formed by coagulation of primary particles smaller than 10 nm with high concentrations, as was observed from the time evolution of number concentrations and size distributions. While concentrations of the lowest size bins were quickly decreasing, concentrations of accumulation mode particles steadily increased.

##### Smelting

Particle size distributions produced by smelting were multimodal. The first mode, <25 nm, was formed by the primary nanoparticles originating from evaporation of the material being smelted and its subsequent condensation. The second mode, around 50 nm, was a product of coagulation of the primary particles. The third mode, with maxima around 200 nm, was most probably coming from the previous operation, MAG welding, where the particles in the workplace did not have enough time to deposit. Most of the particles produced by smelting were released when the smelting oven was open. The measurement after the smelting was finished showed a change of the size distribution shape into unimodal, and the shift of the mode of primary particles to about 25 nm as a product of coagulation. Total median particle number concentration was 4.8 × 10^4^ particles/cm^3^ (IQR 3.1 × 10^4^ to 9.0 × 10^4^ particles/cm^3^), and the highest concentration reached about 2.3 × 10^5^ particles/cm^3^. The time evolution of larger size bins showed a significant contribution of the smallest size bin to the total number concentration for almost the whole sampling period, except the beginning of the measurement, before the smelting vessel was put into the oven.

##### Nanocomposite Machining

Operations of mechanical grinding and milling of various nanocomposites produced primary larger particles >1 µm, but also, nanoparticles produced by condensation in the colder air far from the machining tool of semivolatile organic compounds and thermal breakdown products generated form localized heating of the polymer during machining. In this case, the measured size distribution exhibited unimodal shape, with a maximum at about 100 nm with a large tail for supermicron particles. It seems that these particles may have represented the primary particles produced by grinding and milling. Total median particle number concentration was 5.4 × 10^5^ particles/cm^3^ (IQR 3.1 × 10^5^ to 6.8 × 10^5^ particles/cm^3^), with maximum concentration around 8.8 × 10^5^ particles/cm^3^.

Nevertheless, comparison of the process data with the background measurement in the same workshop floor suggests contribution of other sources of particles. Time plots of the larger size bins revealed that the highest contribution to the total number concentration was from the accumulation mode particles (100 nm–1 µm).

##### Background

All the measurements of the background values showed high particle concentrations, which might have considerably influenced the measurements, making the estimation of the contribution of individual sources very complex. The background values were measured first during the night in the corridor next to the workshops. Then, before each working operation, 15 min of measurements were taken as background values. There were no other obvious sources of aerosol background particles, except from indoor penetration of outdoor particles. Particle number size distributions during the operations are shown in Supplementary Figures S1 and S2.

The percentage of total particulate number concentrations (<10 µm) in larger size bins, measured by SMPS and APS (based on aerodynamic diameter) is shown in Table 2. The highest proportion of particles smaller than 100 nm in diameter was found during machining, whereas the lowest during welding, as can be seen in Table 2. The percentage of total PM number concentrations in larger size bins (<10 µm) is presented in Table 3.

#### 3.2.2. Mass Size Distributions

**Figure 1:**
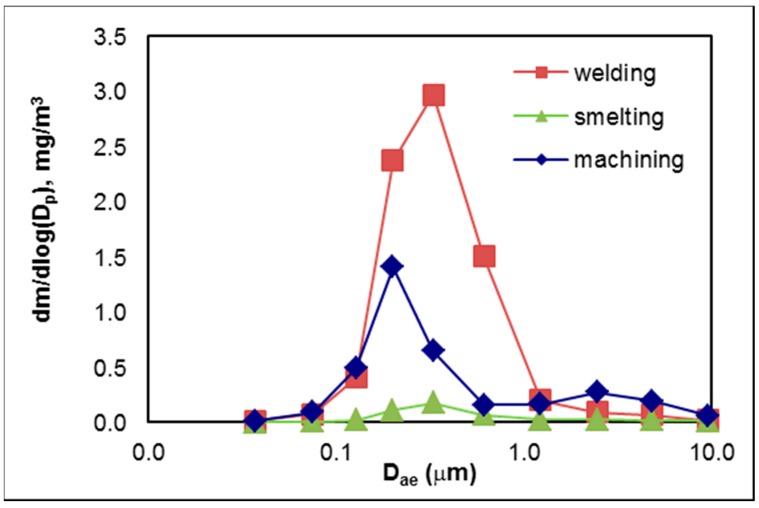
Mass size distribution of aerosol particles produced during welding, smelting, and machining. Values determined by gravimetric analysis of samples collected with the Berner low-pressure cascade impactor (BLPI).

The highest mass concentration measured by BLPI was found during welding, with mean 1.840 mg/m^3^, the second highest during machining, with 0.804 mg/m^3^, and the lowest during smelting, with 0.120 mg/m^3^. Fine mode peaked at around 300 nm in aerodynamic diameter during welding and smelting, when the mass size distributions were almost monomodal, while during machining it peaked at about 200 nm with bimodal mass size distribution, as can be seen in Figure 1.

#### 3.2.3. Size-Resolved Elemental Composition

In the case of welding, the particles were dominated by iron, manganese, and silicon, with manganese and silicon being enriched in comparison with welded material. The maximum of size distribution was between 247 and 435 nm in aerodynamic diameter both for manganese and iron. Silicon concentration was the largest for the biggest analyzed particles.

Smelting operation was done in the same workshop as welding, and despite intensive venting after the previous operation, the analyzed aerosol captured most of signatures of particles from welding. Therefore, the particles were again dominated by iron, manganese, and silicon. Some influence of smelting was visible, especially on last stage, with size range 25–56 nm where aluminum, sodium, and chloride were enriched. The mass size distribution had the same features as from welding. Due to an approximately 30× dilution of aerosol from welding, some influence of ambient aerosol was visible, especially based on sulfur concentrations.

**Figure 2:**
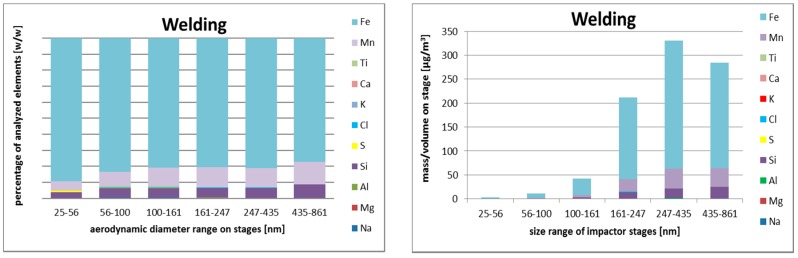
SEM/EDS relative (**left**) and absolute (**right**) elemental composition of size-resolved aerosol fractions during welding, based on samples obtained by Berner low-pressure cascade impactor (BLPI), in the range from 25 nm to 860 nm (six lowest stages, aerodynamic diameter).

**Figure 3:**
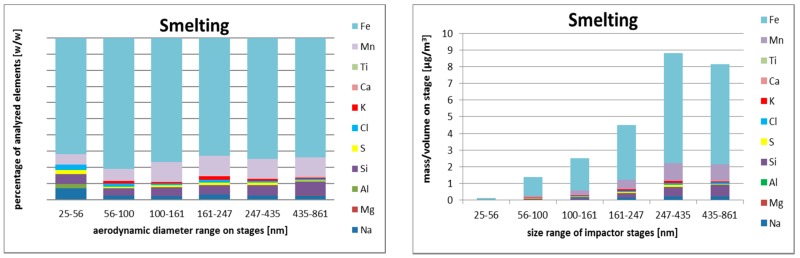
SEM/EDS relative (**left**) and absolute (**right**) elemental composition of size-resolved aerosol fractions during smelting based on samples obtained by Berner low-pressure cascade impactor (BLPI), in the range from 25 nm to 860 nm (six lowest stages, aerodynamic diameter).

**Figure 4:**
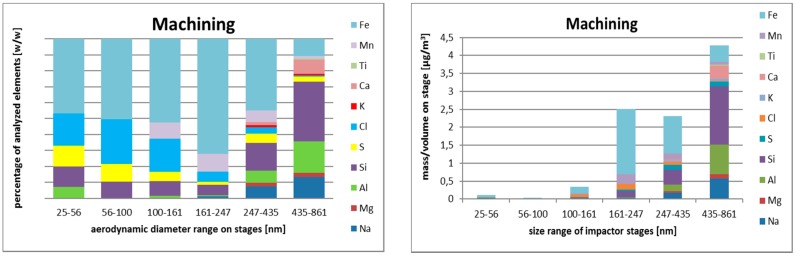
SEM/EDS relative (**left**) and absolute (**right**) elemental composition of size-resolved aerosol fractions during machining based on samples obtained by Berner low-pressure cascade impactor (BLPI), in the range from 25 nm to 860 nm (six lowest stages, aerodynamic diameter).

Elemental composition of size-resolved aerosol fractions during the three operations are shown in Figure 2, Figure 3 and Figure 4.

The data from machining of geopolymers exhibited the largest variability in elemental composition, suggesting a more complex range of sources. Moreover, the total elemental analyzed mass was only a few percent of the total mass, while in previous operations, it accounted for ~50% of the total mass.

We have attempted to calculate the missing mass as a difference between the total mass determined gravimetrically, and sum of all analyzed ions + Al, Si, Ti, Fe, Mn, where analyzed elements were represented by their oxides (Al_2_O_3_, SiO_2_, TiO_2_, Fe_2_O_3_, MnO_2_). Based on our experience from similar past experiments, the missing mass was most probably composed of carbonaceous matter. In the nanofraction, i.e., lowest two impactor stages with particle aerodynamic diameters 25–56 and 56–100 nm, the missing mass formed over 95% of the total mass.

Concerning metals, the smallest particles were rich with iron, chlorine, silicon, and sulfur. Iron and manganese were more abundant in the aerodynamic diameter size range 161–247 nm. The larger particles contained higher concentrations of silicon, aluminum, and sodium. The data in Table 4 show dominance of iron and manganese in first two operations (welding and smelting). 

### 3.3. Oxidative Stress Markers in EBC

In all samples, the markers measured were above the limit of quantitation. No influence of the conductivity on EBC markers’ levels was found. Amylase concentrations in the EBC of all subjects was less than 0.01% of that in saliva [32]. 

No significant decrease was found in EBC markers with increasing latency from last exposure. Similarly, no difference was found between researchers who switched workshops in the previous days or weeks (except 8-isoprostane, that was higher in researchers who continuously worked in workshop 2, *p* < 0.05). 

#### 3.3.1. Markers of Oxidation of Lipids

**Figure 5:**
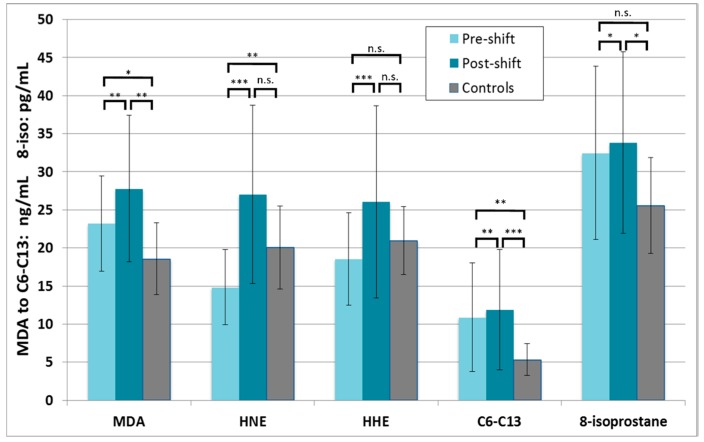
Markers of oxidation of lipids in all 19 nanocomposite workers in pre-shift and post-shift samples) in comparison with controls, * *p* < 0.05, ** *p* < 0.01, *** *p* < 0.001, MDA = malondialdehyde, HNE = 4-hydroxy-*trans*-nonenal, HHE = 4-hydroxy-*trans*-hexenal, C_6_–C_13_ = aldehydes C_6_–C_13_, 8-isoprostane = 8-*iso*-prostaglandin F2α.

The markers of oxidation of lipids in the whole group of researchers are shown in Figure 5. Only pre-shift MDA and aldehydes C_6_–C_13_ were significantly higher in the researchers than controls. However, all post-shift markers derived from the oxidation of lipids, except HHE, were elevated compared to the controls. 

A statistically significant increase was seen for all markers of lipid oxidation in post-shift samples relative to pre-shift: MDA, HNE, HHE, aldehydes C_6_–C_13_, and 8-isoprostane. 

The same markers in the researchers in the subgroups exposed in workshop 1 and workshop 2 are shown in Supplementary Figure S3. The only marker that showed both a pre-shift and post-shift elevation in workshop 2, as compared to workshop 1, was 8-isoprostane; other markers did not differ significantly between the workshops.

#### 3.3.2. Markers of Oxidation of Nucleic Acids and Proteins

**Figure 6:**
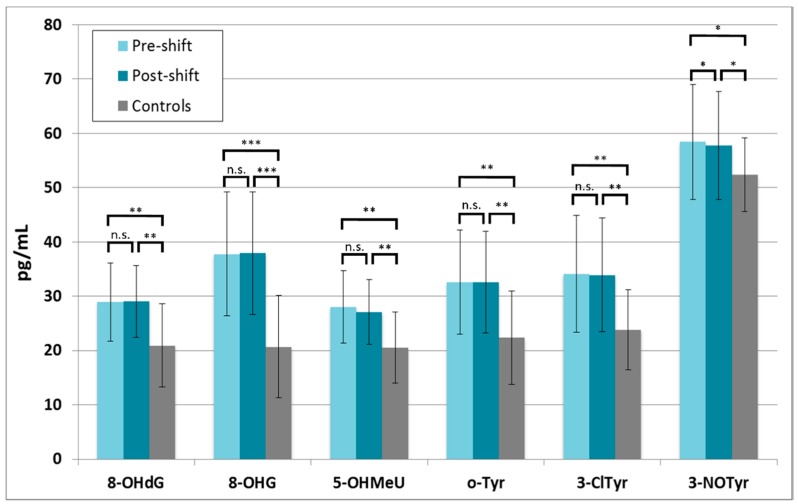
Markers of oxidation of nucleic acids and proteins in all 19 nanocomposites workers in pre-shift and post-shift samples in comparison with the controls, * *p* < 0.05, ** *p* < 0.01, *** *p* < 0.001, 8-OHdG = 8-hydroxy-2-deoxyguanosine, 8-OHG = 8-hydroxyguanosine, 5-OHMeU = 5-hydroxymethyl uracil, *o*-Tyr = *o*-tyrosine, 3-ClTyr = 3-chlorotyrosine, 3-NOTyr = 3-nitrotyrosine.

In contrast to lipid oxidation markers, all markers of oxidation of nucleic acids and proteins were already significantly elevated in the pre-shift EBC samples, and no further increase was observed in post-shift EBC samples (Figure 6). The same markers in the researchers in the subgroups exposed in workshop 1 and workshop 2 are shown in Supplementary Figure S4. No significant differences between the workshops were seen.

#### 3.3.3. Correlations of Markers with Exposure and Symptoms 

Two markers, namely 5-OHMeU and *o*-Tyr, were correlated with the years of employment in these operations. Post-shift 3-NOTyr correlated with chronic bronchitis, defined as chronic, productive cough for three months in each of two consecutive years [33], as can be seen in Table 5.

#### 3.3.4. Correlations of the Levels of Markers in the Pre-Shift and Post-Shift Samples

The level of the majority of markers in the pre-shift collection correlated with the identical markers in the post-shift samples. In addition, the level of a large number of biomarkers correlated with the level of other biomarkers in the same or in the other samples, as presented in Supplementary Table S2.

#### 3.3.5. Association of EBC Markers with Occupational Exposure

Multiple regression analysis confirmed a significant association (*p* < 0.05) between production and machining of nanocomposites in nine pre-shift and nine post-shift markers, as shown in Table 6 and Table 7. Non-occupational factors were generally not significantly associated with EBC biomarkers.

### 3.4. Environmental Contamination

The level of air pollution with SO_2_, NO*_x_*, O_3_, PM_2.5_, and PM_10_ at both sites was classified by the National Hydrometeorological monitoring system as very low or low. The values did not exceed the recommended limits. No positive correlation of both pre-shift and post-shift markers of oxidative stress with environmental levels was seen. 

## 4. Discussion

A large number of nanomaterials has been found in experimental studies to induce toxicity mediated by reactive oxygen species in many biological systems [19]. Reactive oxygen species can attack lipids, nucleic acids, proteins, and further essential biomolecules. This process finally damages mitochondrial structure, causes depolarization of mitochondrial membrane, impairment of the electron transport chain, and the activation of the NADPH-like system. Reduced levels of antioxidants then lead to cellular injury or death by the alteration of signaling pathways. DNA injuries, with single-strand and double-strand breakages were seen, with consequent cell cycle arrest or apoptosis [34,35]. Obviously, these processes are not specific to exposure to nanomaterials.

Although production of the nanocomposites in this research plant included the addition of nanoparticles (nanoSiO_2_), the main proportion of nanoparticles in workplace aerosol originated during machining and smelting of the samples using abrasive structures to modify the surface of the products or at hot temperature. Iron represented the highest proportion of elements detected in the aerosol during all operations monitored, including machining. Similarly, when the elements were ranked according to their mass concentrations in the nanosized fractions (last two impactor stages), Fe was the more abundant element, followed by Mn, Si, Al, S, Na, Cl, and K. This means that even though nanoSiO_2_ was added in substantial amounts into the machined polymeric materials, its release into the form of nanosized aerosol particles was not as significant. We presume that most of the aerosol mass during machining operation was likely composed of organic compounds, as shown in several of our previous studies [1,2,4], which was not quantifiable in SEM/EDS analysis. When elements are ranked according to their summed mass concentrations over the last six stages of the impactor (from 25 to 860 nm), the elements were present in the following descending order of abundance: Fe, Mn, Si, Na, S, Cl, Al, Ca, K, Mg, and Ti, for all three operations. Elements other than iron originated mostly from geopolymers and epoxide resins during machining of the surface. It has been shown that composite synthesis generates semivolatile organic compounds, especially related to additives in the polymer [3,4,36]. In regard to exposures, our findings are broadly consistent with this prior work. Of course, other substances that are specifically toxic and generated in combustion processes like welding and smelting, including e.g., ozone, may have substantially contributed to the analyzed effects. 

Exposure to airborne aerosols in this sector are to mixtures. Even though some Fe oxides are safe for specific biomedical applications, other uses need to be considered more carefully. The adverse toxicological outcomes across different testing platforms are not consistent, and it seems that the nanoparticle coating and protein corona effects may alter nanoparticle dissolution rate, as well as clearance and translocation biokinetics, and make them less toxic [37]. This, however, is not the case in the manufacturing and machining of nanocomposites containing metal oxides, including Fe oxides. 

The results of elevated markers of oxidative stress agree with our previous findings in other production plants, where workers were exposed to poorly soluble engineered metal oxide nanoparticles. The highest levels of lipids, nucleic acids, and protein markers of oxidative stress reached about 130% of the values reported here [38]. In those nanoTiO_2_ production workers, median mass and number concentrations were the highest (0.65 mg/m^3^ and 2.3 × 10^4^ particles/cm^3^, respectively) and exposures in the workshop were longer (3.5 h/day, with the rest of the shifts spent in the neighboring operating rooms with no exposure to nanoTiO_2_) [39]. The length of the shift was also longer in Fe oxide manufacturing plants, with median gravimetric and number concentrations of 0.083 mg/m^3^ and 6.7 × 10^4^ particles/cm^3^, respectively. The results of most markers of oxidation of lipids, nucleic acids, and proteins in the Fe oxide study were 110–120% higher than in nanocomposite researchers [40]. 

The aerosol mass and number concentrations were higher in this study of nanocomposite workers than in our earlier studies of nanoTiO_2_ and iron oxide manufacturing workers, but the duration of the operations associated with nanoparticle exposures was shorter, typically <2 h/day, with the remainder of the shift spent in offices in another part of the building. Another important consideration is that in previous factories, the proportion of the nano-sized particles in the inhaled aerosol was high (80–85%), whereas in the current study, it was lower and more variable (40–95%). Even in the office, in the employees who visited the TiO_2_ production area and were exposed to the aerosol for an average of 14 min/day, the EBC markers reached 50–60% of the levels measured in TiO_2_ production workers, which was significantly higher when compared to controls [41,42]. 

No association was seen with lifestyle factors or environmental air contamination in any of the studies, including this one.

Of note, EBC markers of oxidative stress in this study were already elevated in pre-shift samples, suggesting chronic effects. Accordingly, in our previous study, the pre-shift markers were elevated [38], and TiO_2_ particles were identified in the pre-shift EBC samples of the workers [43]. The biokinetics of tissue recovery from previous injury in humans, in the absence of further exposure, is not well documented in these cohorts of workers, but it is likely that these effects can last for months to years. In a short inhalational study using 10 mg/m^3^ nanoTiO_2_ in rats over 2 weeks (6 h/day, 5 days/week), histopathological lesions persisted at post-exposure day 7, but resolved at day 15 [44]. 

Furthermore, other human studies brought results supporting the potential of nanoparticles to produce oxidative stress in human volunteers [45,46,47,48,49]. Current research on health effects and epidemiology of nanomanufacturing workers, focusing on the respiratory system and its closely associated cardiovascular system and circulation, is limited. However, Liou et al. [50,51] recently found significantly higher 8-isoprostane in EBC and 8-OHdG in the urine of workers with occupational exposure to metal oxide nanomaterials (TiO_2_, SiO_2_, or indium tin oxide). In a recent study of workers exposed to high levels of nanoTiO_2_, Zhao et al. (2018) documented elevated cardiopulmonary disease markers, impairment of lung function, X-ray interstitial changes, and elevated blood MDA and TNF biomarkers that were associated with occupational exposure [52]. In the current group of nanocomposite researchers, we have found borderline post-shift spirometry impairments, more frequent chronic bronchitis, as well as increased pro-inflammatory markers of lung injury, such as cytokines and leukotrienes [24]. This is also in agreement with the results in employees of photocopy workstations [53]. 

One major limitation of this study is the small number of subjects, which unfortunately, is limited by the small size of this workforce, as all available research workers were included. This is a common issue in epidemiological studies of nanomanufacturing workers because the industry is still evolving, consolidating, and changing all the time. Secondly, aerosol measurements were based on area sampling, not personal exposures, as good quality personal nanoparticle samplers were not available at the time of this field study. Another challenge is the mixed nature of exposures, which makes it difficult to discern effects resulting from engineered nanoparticles, incidental ones, nanocomposite synthesis and processing, or auxiliary operations such as welding. As has already been mentioned, other substances specifically generated in combustion processes, such as welding and smelting, like e.g., ozone, carbon monoxide, and polycyclic aromatic hydrocarbon, are likely to be causal for the effects analyzed. 

Due to limited access to the workplace, we could only collect a relatively small number of measurements. We acknowledge limited characterization of exposure variability in the current study. Although aerosol exposures were well characterized for each operation, day-to-day and seasonal variability due to variations in operations, working conditions, and activities, requires repeated measures. Furthermore, we do not have a good explanation for the high background exposure levels measured in some cases, although persisting nanoparticles in background air from past working operations cannot be excluded. This study is ongoing, and these limitations will be addressed in follow-up visits. Exposure sources in individual workshops/laboratories were usually mixed, and it is difficult to apportion aerosol source contribution to the overall nanoparticle exposures. 

A notable strength of our study is the large spectrum of biomarkers and clinical endpoints that were examined. Consistency in study design and biomarker endpoints across several studies of nanoparticle-exposed workers further enabled us to examine relationships between pre-shift and post-shift biomarkers in EBC, and identify the more robust biomarkers for biomonitoring purposes. Due to frequent correlation between the pre-shift and post-shift markers in this study, we could limit EBC collection to the post-shift samples. Furthermore, by exploring the patterns of biomarker from our earlier studies and the strength of association in multiple regression analysis, we could narrow the broad spectrum of markers of oxidation to the following markers that appeared to be the most robust set of markers across all our studies: 8-OHdG and 5-OHMeU from nucleic acids; *o*-Tyr and 3-NOTyr from proteins; and MDA, and ALD6-12 from lipids. We will also keep 8-isoprostane, a biomarker that differentiated the subgroups of researchers (higher in workshop 2 during machining of nanocomposites) in subsequent investigations. 

One major challenge with sensitive biomonitoring techniques is their limited specificity towards exposure triggers and the difficulty in interpreting the meaning of their physiological values in the context of chronic disease development and damage-repair kinetics. It is likely the transition metals, especially Fe and Mn and their dissolved ions, contribute significantly to oxidative stress in lung tissues [15,34]. Similarly elevated oxidative stress markers in EBC have been seen in patients exposed to asbestos and silica [54,55,56]. The contribution of organics and biopersistent polymeric particles in oxidative stress and inflammatory responses, in this cohort of workers, cannot be ruled out.

## 5. Conclusions

Our results are consistent with the oxidative stress hypothesis, and suggest lung injury at the molecular level. Researchers in nanocomposite processing had elevated levels of oxidative stress markers in EBC. In a companion manuscript, we also report on elevated levels of inflammatory markers in EBC and chronic bronchitis among this cohort of workers. These effects are likely caused by mixed exposures to aerosols, originating from welding, smelting, and nanocomposite processing. 

More research is needed, as other substances generated in combustion processes, including welding and smelting, like e.g., ozone, or polycyclic aromatic hydrocarbons in both workshops, should be evaluated. 

Considering that exposures in this study were long-term (close to two decades), but of short duration and frequency (no longer than 2 h/day in total, and often shorter), chronicity of such short exposure episodes is sufficient to trigger chronic oxidative stress and inflammatory responses comparable to the levels of EBC biomarkers found in (nano)Fe oxide nanomanufacturing workers, and of the same order of magnitude (although lower) than in nanoTiO_2_ manufacturing workers who were exposed for longer operation durations over similar periods of time. 

We can strongly recommend using both the ventilation system in the workshops and the personal protection equipment of the researchers. 

Analysis of exhaled breath has immense potential to bring data enabling monitoring of several exposures from the workplace and the environment where nanoparticles and other inhalable toxicants may play a significant role. We recommend post-shift analysis of the following markers: 8-OHdG, 5-OHMeU, *o*-Tyr, 3-NOTyr, MDA, ALD6-12, and 8-isoprostane as the most sensitive and robust biomarkers across all our EBC studies.

## Figures and Tables

**Table 1:** Characteristics of the groups of subjects.

Subjects	Exposed	Controls
Number of Subjects	19	19
**Age (years)**	42.4 ± 11.4	45.4 ± 11.7
**Body Mass Index (kg/m^2^)**	27.69 ± 6.30	24.39 ± 4.04
**Alcohol occasionally (*n*, %)**	17 (90%)	16 (84%)
**Employment in nanocomposite production (years)**	Mean 18.0 ± 10.3	-

**Table 2:** Summary statistics of the number concentration and size distribution of the aerosol during various operations. TNC = total number concentration.

Operation	*N* (Number of SMPS/APS Samples)	TNC (particles/cm^3^), Median	TNC, Maximum	Size Distribution (Main Mode)	% of Particles <100 nm	Ratio Operation/Background	Mass Concentration (mg/m^3^)
**Smelting**	32	4.8 × 10^4^	2.0 × 10^5^	<25 nm	95	2.3	0.120
**Machining**	39	5.4 × 10^5^	8.2 × 10^5^	100 nm	61	1.9	0.804
**Welding**	28	1.3 × 10^5^	2.5 × 10^5^	200 nm	40	6.2	1.840
**Background smelting/welding**	5	2.1 × 10^4^	2.5 × 10^4^	<10 nm	97	1	not measured
**Background machining**	5	2.8 × 10^5^	3.4 × 10^5^	130 nm	41	1	not measured

**Table 3:** Percentage of total PM number concentrations (<10 µm) in larger size bins measured by SMPS and APS (based on aerodynamic diameter).

Percentage from Total <10 µm		<25–100 nm		100 nm–10 µm				
		<25 nm	25–100 nm	100 nm–1 µm	1–2.5 µm	2.5–10 µm	Total <1 µm	1–10 µm
**Basement**	**Metal Active Gas (MAG) Welding**	3.35	36.78	59.85	0.02	0.00	99.97	0.03
	**Smelting**	69.64	25.00	5.36	0.01	0.00	99.99	0.01
	**Background—15 min before welding**	74.37	22.39	3.23	0.00	0.00	99.99	0.01
**Ground floor**	**Machining (Milling & Grinding)**	2.61	58.62	38.76	0.00	0.00	99.99	0.01
	**Background—15 min before machining**	0.27	40.62	59.10	0.01	0.00	99.99	0.01
**Background-night—15 h**		4.24	66.40	29.32	0.04	0.00	99.96	0.04

**Table 4:** Elemental mass concentration during various operations and diagnostic ratios.

Operation	Mass Concentration (µg/m^3^)						Diagnostic Ratios	
	Fe	Mn	Ti	Ca	Si	Mg	Mn/Fe	Si/Fe
**Welding**	703	114	0	0	59	0	0.16	0.08
**Smelting**	19	3.00	0	0.04	1.70	0.01	0.16	0.09
**Machining**	3.60	0.55	0.03	0.42	2.20	0.17	0.16	0.63

**Table 5:** Correlations of pre-shift and post-shift exhaled breath condensate (EBC) markers with the length of exposure and chronic bronchitis in the researchers exposed to nanocomposites.

	Pre-Shift Marker, Correlation Coefficient (*p* Value)	Post-Shift Marker, Correlation Coefficient (*p* Value)
**Employment in nanocomposite production (years)**	5-OHMeU, 0.477 (0.039) *o*-Tyr, 0.488 (0.034)	- *o*-Tyr, 0.511 (0.025)
**Chronic bronchitis**	-	3-NOTyr, 0.496 (0.031)

**Table 6:** Multiple regression analysis (regression coefficient and 95% CI) of nanocomposite exposure, age, gender, alcohol, body mass index (BMI), and pre-shift oxidative stress markers in the exhaled breath condensate. C_6_–C_13_ = aldehydes C_6_–C_13_, 8-OHdG = 8-hydroxy-2-deoxyguanosine, 8-OHG = 8-hydroxyguanosine, 5-OHMeU = 5-hydroxymethyl uracil, *o*-Tyr = *o*-tyrosine, 3-ClTyr = 3-chlorotyrosine, 3-NOTyr = 3-nitrotyrosine. * *p* < 0.05, ** *p* < 0.01, *** *p* < 0.001.

Pre-Shift Markers	MDA	C_6_–C_13_	8-isoprostane	8-OHdG	8-OHG	5-OHMeU	*o*-Tyr	3-ClTyr	3-NOTyr
**Nanocomposites production (Yes/No)**	**4.71** * (1.19, 8.23)	**7.06** *** (3.58, 10.54)	**8.02** * (1.36, 14.69)	**8.61** ** (3.26, 13.97)	**17.66** *** (9.97, 23.35)	**7.46** ** (2.82, 12.09)	**10.12** ** (3.64, 16.59)	**11.31** *** (5.00, 17.61)	**6.74** * (0.36, 13.12)
**Age (years)**	**−0.23** ** (−0.40, −0.70)	0.53 (−0.11, 0.22)	0.03 (−2.29, 0.34)	0.10 (−0.15, 0.35)	0.11 (−0.25, 0.47)	0.18 (−0.04, 0.39)	0.17 (−0.13, 0.47)	**0.34** * (0.44, 0.63)	−0.02 (−0.31, 0.28)
**Gender (Male/Female)**	0.73 (−3.56, 5.01)	−0.84 (−5.07, 3.40)	0.10 (−8.01, 8.21)	4.00 (−2.51, 10.52)	1.96 (−7.40, 11.31)	2.38 (−3.27, 8.02)	5.61 (−2.26, 13.49)	3.92 (−3.76, 11.60)	4.34 (−3.42, 12.10)
**Alcohol (Yes/No)**	−1.53 (−7.18, 4.11)	2.61 (−2.97, 8.18)	3.79 (−6.89, 14.47)	3.73 (−4.85, 12.31)	3.50 (−8.83, 15.82)	4.59 (−2.84, 12.03)	4.63 (−5.74, 15.00)	2.72 (−7.39, 12.83)	2.73 (−7.49, 12.94)
**BMI (kg/m^2^)**	−0.83 (−0.45, 0.28)	−0.38 * (−0.74, −0.20)	−0.28 (−0.97, 0.40)	−0.25 (−0.80, 0.31)	−0.18 (−0.97, 0.62)	0.26 (−0.45, 0.51)	−0.07 (−0.74, 0.60)	−0.37 (−1.02, 0.28)	−0.30 (−0.96, 0.36)

**Table 7:** Multiple regression analysis (regression coefficient and 95% CI) of nanocomposite exposure, age, gender, alcohol, body mass index (BMI), and post-shift markers of oxidation in the exhaled breath condensate. MDA = malondialdehyde, HNE = 4-hydroxy-*trans*-nonenal, C_6_–C_13_ = aldehydes C_6_–C_13_, 8-isoprostane = 8-*iso*-prostaglandin F2α, 8-OHdG = 8-hydroxy-2-deoxyguanosine, 8-OHG = 8-hydroxyguanosine, 5-OHMeU = 5-hydroxymethyl uracil, *o*-Tyr = *o*-tyrosine, 3-ClTyr = 3-chlorotyrosine. * *p* < 0.05, ** *p* < 0.01, *** *p* < 0.001.

Post-Shift Markers	MDA	HNE	C_6_–C_13_	8-isoprostane	8-OHdG	8-OHG	5-OHMeU	o-Tyr	3-ClTyr
**Nanocomposites production (Yes/No)**	**8.71** ** (4.33, 14.00)	**8.48** ** (2.20, 14.75)	**8.27** *** (4.47, 12.08)	**9.37** ** (2.44, 16.31*)*	**8.23** ** (3.00, 13.47)	**17.78** *** (10.12, 25.43)	**5.74** ** (1.21, 10.27)	**10.27** ** (3.88, 16.65)	**11.09** *** (4.88, 17.31)
**Age (years)**	−0.20 (−0.45, 0.49)	0.18 (−0.28, 0.31)	0.05 (−0.13, 0.23)	0.05 (−0.28, 0.37)	−0.05 (−0.29, 0.20)	0.11 (−0.25, 0.47)	−0.03 (−0.24, 0.19)	0.17 (−0.13, 0.47)	**0.33** * (0.04, 0.62)
**Gender (Male/Female)**	2.33 (−4.10, 8.76)	6.06 (−1.58, 13.70)	−0.66 (−5.29, 4.00)	0.18 (−8.26, 8.62)	2.09 (−4.28, 8.45)	1.70 (−7.62, 11.01)	1.55 (−3.96, 7.06)	5.52 (−2.25, 13.29)	3.73 (−3.84, 11.29)
**Alcohol (Yes/No)**	−0.88 (−9.34, 7.58)	2.15 (−7.91, 12,21)	2.81 (−3.28, 8.90)	3.10 (−8.01, 14.21)	1.34 (−7.05, 9.72)	3.32 (−8.94, 15.59)	1.79 (−5.47, 9.05)	4.75 (−5.48, 14.98)	2.63 (−7.34, 12.59)
**BMI (kg/m^2^)**	0.06 (−0.49, 0.60)	−0.61 (−1.25, 0.42)	**−0.45** * (−0.84, −0.05)	−0.29 (−1.01, 0.43)	−0.09 (−0.63, 0.45)	−0.16 (−0.95, 0.63)	0.23 (−0.24, 0.70)	−0.93 (−0.75, 0.57)	0.36 (−1.01, 0.28)

## Supplementary Materials

The following are available online at http://www.mdpi.com/2079-4991/8/8/611/s1.
