# Supplementary material for: Markers of Oxidative Stress in the Exhaled Breath Condensate of Workers Handling Nanocomposites
Source: Nanomaterials (Basel). 2018 Aug 10;8(8):611. doi: 10.3390/nano8080611 (PMC6116291; doi:10.3390/nano8080611)
Supplement: Supplementary file 1 [file nanomaterials-08-00611-s001.pdf]

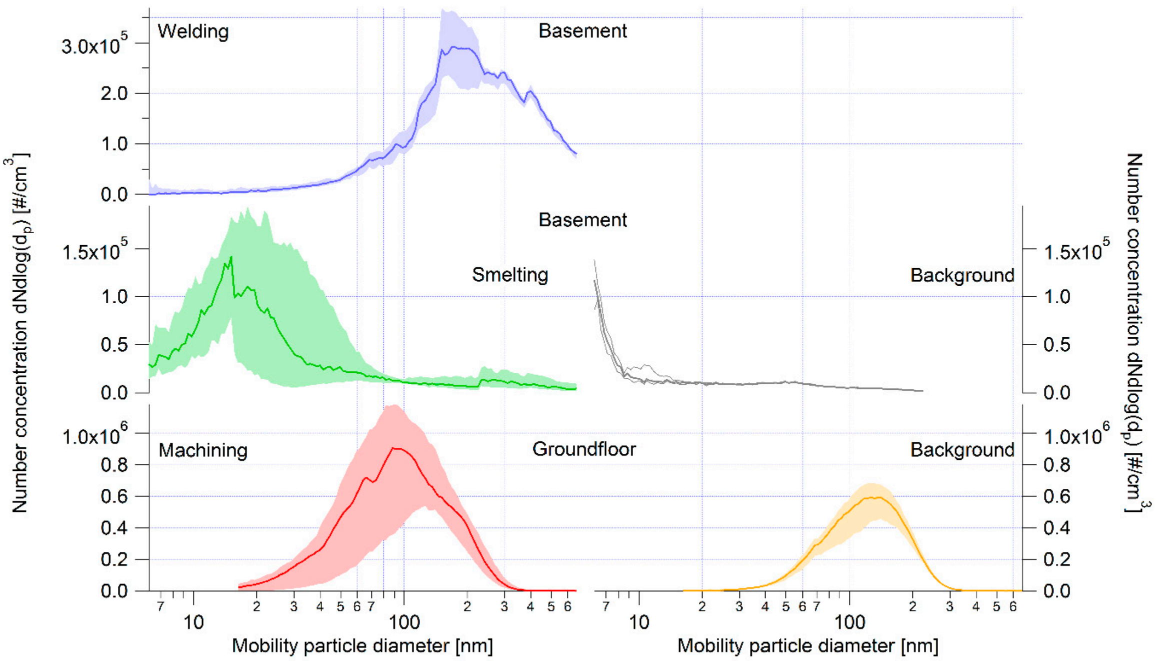

Figure S1. Particle number size distributions during welding, smelting, and machining (grinding and milling), and corresponding backgrounds measured by SMPS; solid line represents median for the measurement period and the corresponding shaded area shows interquartile span.

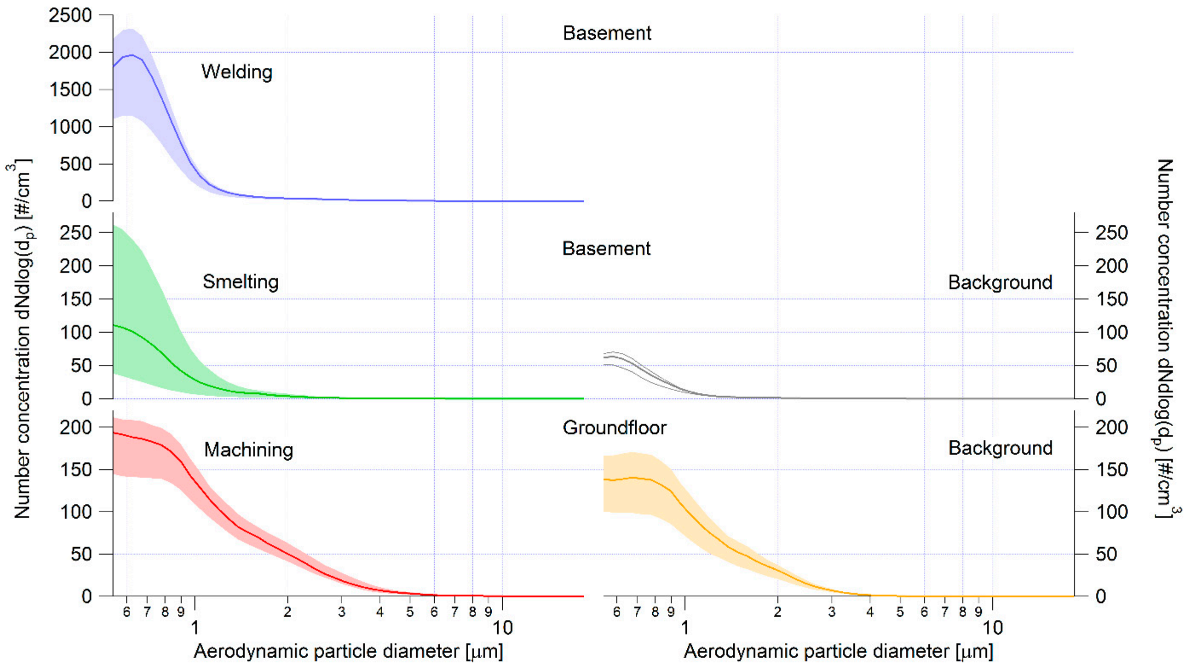

Figure S2. Particle number size distributions during welding, smelting, and machining (grinding and milling), and corresponding backgrounds measured by APS; solid line represents median for the measurement period and the corresponding shaded area shows interquartile span.

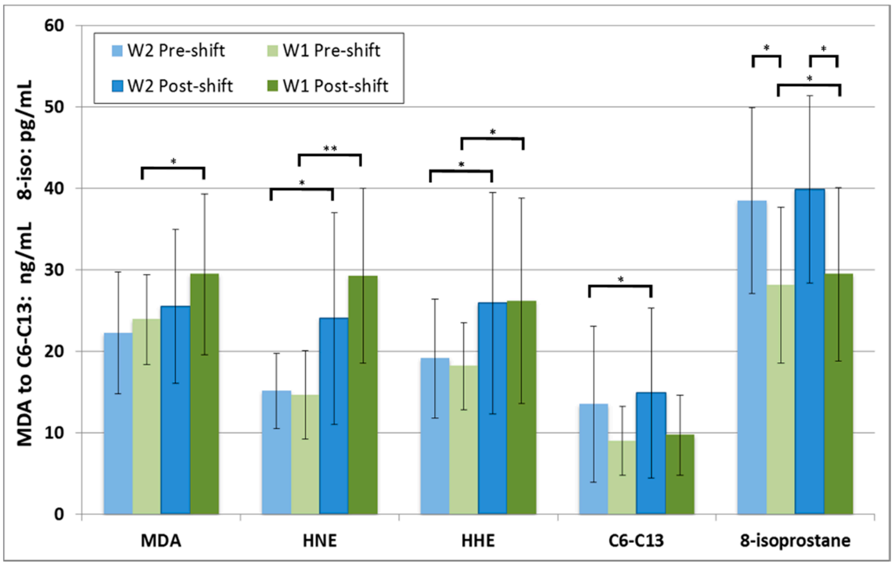

Figure S3. Markers of oxidation of lipids in the subgroups of 11 nanocomposites workers from workshop 1 (welding and smelting) and 8 from workshop 2 (machining) in pre-shift and post-shift samples). *(p<0.05) **(p<0.01) MDA=malondialdehyde, HNE=4-hydroxy-trans-nonenal, HHE=4-hydroxy-trans-hexenal, C6-13 =aldehydes C6-C13, 8-isoprostane=8-isoProstaglandin F2α.

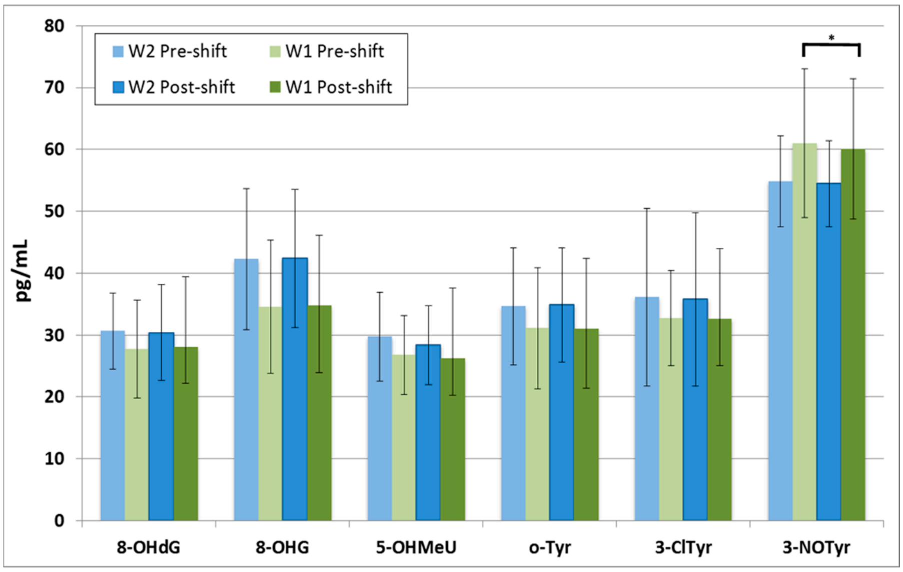

Figure S4. Markers of oxidation of nucleic acids and proteins in the subgroups of 11 nanocomposites workers from workshop 1 (welding and smelting) and 8 from workshop 2 (machining) in pre-shift and post-shift samples. *(p<0.05), 8-OHdG=8-hydroxy-2-deoxyguanosine, 8-OHG=8-hydroxyguanosine, 5-OHMeU=5-hydroxymethyl uracil, o-Tyr=o-tyrosine, 3-ClTyr=3-chlorotyrosine, 3-NOTyr=3-nitrotyrosine.

Table S1. Number of aerosol samples collected in the workshops.

Note: The online instruments were placed about 1.5 m from the workers in the height of approximately 1.5 m. The impactors were placed about 3 m from the individual processes in the height of approximately 1.5 m.

**Process**	**Number of samples**	
	SMPS/APS	Impactor
Workshop 1 -Welding	28	1
Workshop 1 - Smelting	32	1
Workshop 2 - Machining	39	1
Workshop 1 Background	5	0
Workshop 2 Background	5	0

Table S2. Correlations of the levels of different marker in the pre-shift or post-shift samples.

	**Correlation**	**p-value**
**Pre-shift markers with other Pre-shift markers**		
1 MDA with 1 C6-C13	0.474	0.040
1MDA with 1 5-OHMeU	-0.610	0.006
1 HNE with 1 HHE	0.514	0.024
1 HHE with 1 8OHG	0.501	0.029
1 8-isoprostane with 1 C6-C13	0.460	0.047
1 8-OHdG with 1 8OHG	0.800	0.000
1 8-OHdG with 1 5-OHMeU	0.761	0.000
1 8-OHdG with 1 o-Tyr	0.686	0.001
1 8-OHdG with 1 3-ClTyr	0.701	0.000
1 8 OHG with 1 5-OHMeU	0.563	0.012
1 8-OHG with 1 o-Tyr	0.634	0.004
1 8-OHG with 1 3-ClTyr	0.799	0.000
1 5-OHMeU with 1 o-Tyr	0.820	0.000
1 5-OHMeU with 1 3-ClTyr	0.692	0.001
1 o-Tyr with 1 3-ClTyr	0.625	0.004
**Pre-shift markers with other Post-shift markers**		
2-HNE with 1 MDA	0.715	0.001
2 HNE with 1 HHE	0.594	0.007
2-HHE with 1-MDA	0.578	0.010
2-HHE with 1 8-OHG	0.615	0.005
2 C6-C13 with 1 MDA	0.478	0.038
2 C6-C13 with 1 8-isoprostane	0.460	0.047
2 8-isoprostane with 1 C6-C13	0.493	0.032
2 8-OHdG with 1 HHE	-0.482	0.037
2 8-OHG with 1 HHE	0.486	0.035
2 8-OHG with 1 8-OHdG	0.794	0.000
2 8-OHG with 1 5-OHMeU	0.564	0.012
2 8-OHG with 1 o-Tyr	0.645	0.003
2 8-OHG with 1 3-ClTyr	0.796	0.000
2 5-OHMeU with 1 HHE	-0.470	0.042
2 o-Tyr with 1 8-OHdG	0.689	0.001
2 o-Tyr with 1 8-OHG	0.628	0.004

2 o-Tyr with 1 5-OHMeU	0.846	0.012
2 o-Tyr with 1 3-ClTyr	0.642	0.003
2 3-ClTyr with 1 8-OHdG	0.696	0.001
2 3-ClTyr with 1 8-OHG	0.783	0.000
2 3-ClTyr with 1 5-OHMeu	0.706	0.001
2 3-ClTyr with 1 o-Tyr	0.628	0.004
**Post-shift markers with other Post-shift markers**		
2 MDA with 2 HNE	0.790	0.000
2 MDA with 2 HHE	0.684	0.001
2 HNE with 2 HHE	0.790	0.000
2 HHE with 2 8-OHG	0.604	0.006
2 HHE with 2 5-OHMeU	-0.476	0.039
2 C6-C13 with 8-isoprostane	0.488	0.034
2 8-OHdG with 2 5-OHMeU	0.809	0.000
2 OHG with 2 o-Tyr	0.642	0.003
2 OHG with 2 3-ClTyr	0.781	0.000
2 o-Tyr with 2 3-ClTyr	0.648	0.003
